# Acute Traumatic Cataract Diagnosed by Ocular Point of Care Ultrasound (POCUS) in the Emergency Department

**DOI:** 10.24908/pocusj.v10i01.18110

**Published:** 2025-04-15

**Authors:** Adrian Huffard, Shannon Overholt, Angelo Kantrales, Taryn Hoffman

**Affiliations:** 1 HCA Florida Orange Park Hospital, Orange Park, FL, USA

**Keywords:** ocular ultrasound, trauma, emergency medicine, emergency ultrasound

## Abstract

**Introduction:**

It is estimated that over 55 million people suffer ocular injuries each year. Of these injuries, approximately 1.6 million are found to suffer permanent visual impairment secondary to traumatic cataract. Although a traumatic cataract can be a vision threatening pathology, it may be overlooked or difficult to diagnose. The objective of this report is to demonstrate the utility of ocular point of care ultrasound (POCUS) in the emergency department while highlighting its potential to diagnose a traumatic cataract. Case

**Report:**

A 66-year-old man presented to the emergency department with suspected cervical spine injury after being involved in a bicycle accident. During the secondary survey, the patient developed sudden painless loss of vision in his left eye. Computed tomography (CT) and external ocular exam did not reveal the cause of his vision loss. Emergency physicians employed the use of point of care ultrasound POCUS to diagnose an acute traumatic cataract as the etiology, which was later confirmed by Ophthalmology.

**Conclusion:**

With the adoption of ocular POCUS as a staple in emergency medicine residency training, this case is testimony to its growing functionality in the setting of ocular trauma. We pose that it may aid as a diagnostic tool, avoid gratuitous testing, and ultimately expedite specialist evaluation and definitive treatment.

## Introduction

Cataract diagnosis and classification is traditionally made under direct ophthalmoscopy or, under ideal circumstances, dilated slit lamp exam. However, a complete and accurate exam is often impractical in the setting of ocular trauma. Primary emergency department examination may be limited not only by physician expertise and resource availability, but also by associated injuries [1,2]. Even in instances where physicians are able to rule out concomitant ocular injuries with advanced imaging, prompt and reliable evaluation of lens integrity is of critical importance. Ocular ultrasound (US) has proven to be a useful tool for initial diagnosis of suspected traumatic cataract and other ocular injuries [3]. Given the widespread use of point of care ultrasound (POCUS) by trained emergency medicine physicians, we advocate for incorporating ocular POCUS to enhance diagnostic accuracy and expedite care in cases of acute orbital trauma. In this report, we describe a case in which ocular POCUS was used to make the diagnosis of acute traumatic cataract in a patient who complained of unilateral vision loss after blunt trauma.

## Case Report

A 66-year-old man presented to the emergency department as a level one trauma activation following a bicycle accident. He had dynamic weakness of upper extremities and was ultimately found to have a cervical spinal cord injury. Following primary survey and computed tomography (CT), the patient developed sudden painless vision loss of the left eye. Workup to that point had been negative for orbital fracture, entrapment, retrobulbar hematoma, tethering of the optic nerve, lens dislocation, penetrating globe injury, or overt signs of globe rupture.

**Figure 1.**
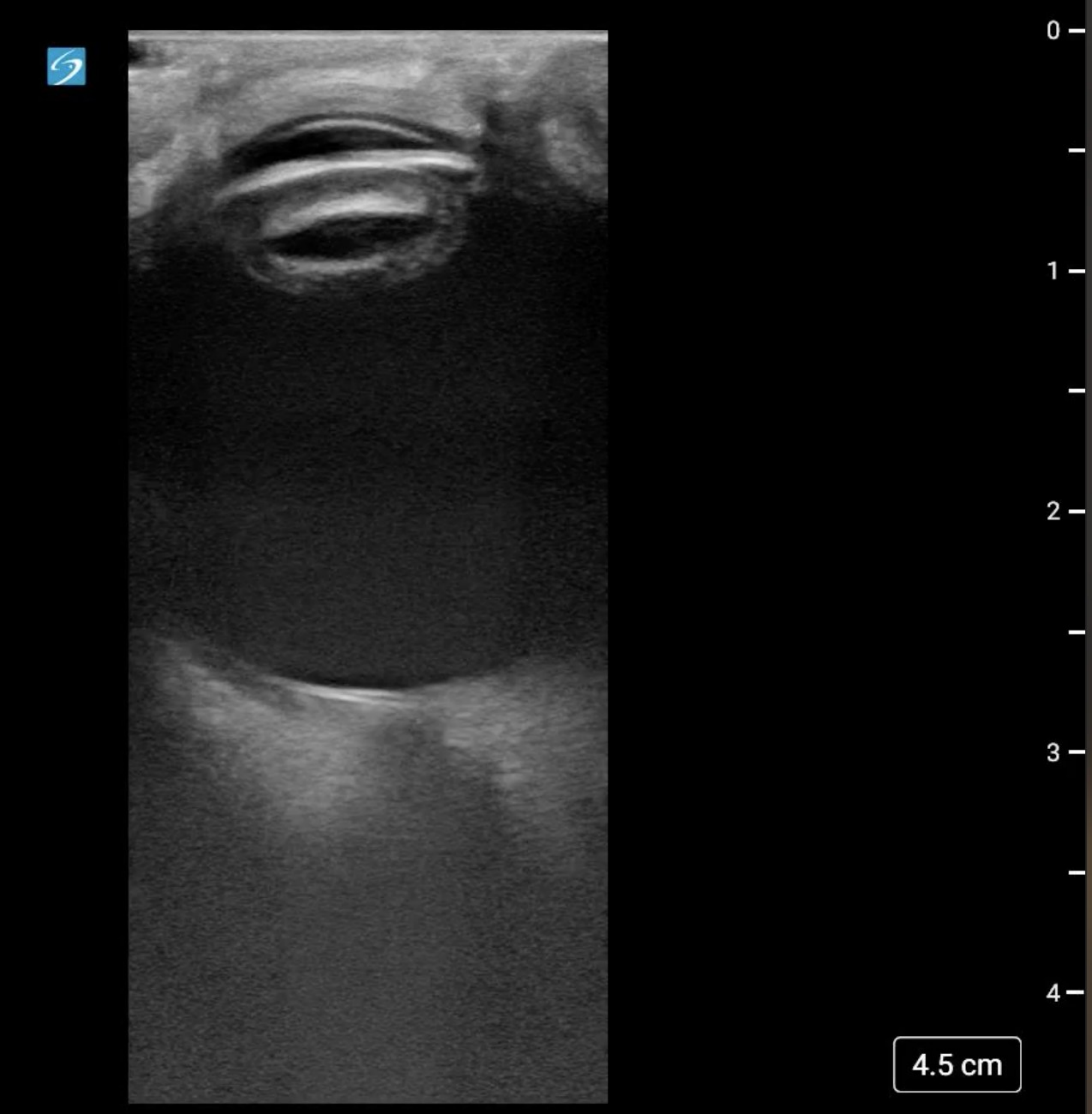
Ocular point of care ultrasound (POCUS) image of the patient's affected eye. Lens is irregular and thickened with increased echogenicity.

Considering potential underlying vitreous hemorrhage, retinal detachment, lens dislocation, or traumatic cataract, an ocular POCUS was performed by the emergency physician, which demonstrated an irregular, thickened lens with increased echogenicity, consistent with cataract (Figure 1). With this working diagnosis, a formal ophthalmology consultation was requested in the emergency department. The ophthalmologist performed a detailed ocular examination and agreed with the diagnosis of acute traumatic cataract.

## Discussion

Traumatic cataracts may occur secondary to penetrating or blunt trauma as well as electrical shock, radiation, and caustic exposure [3]. If suspected, cataracts may be diagnosed under direct ophthalmoscopic exam, however, a complete exam requires a dilated slit lamp exam. Slit lamp exam is the gold standard and can be used to assess the type and severity of cataract and to recognize concomitant injuries. Potential acuity meter, US, and glare/contrast sensitivity testing may also aid in diagnosis, prognostication, and presurgical planning [4].

Acute traumatic cataracts may be overlooked or missed on the first presentation. Even in instances when a patient complains of acute monocular vision loss, adequate and complete ocular exam is infrequently feasible or fruitful. Primary exam may be limited by concomitant injuries and associated deformities. Corneal lacerations, lens dislocation, hyphema, soft tissue contusion, and facial fractures can directly obstruct lens visualization during direct ophthalmoscopy. In addition, trauma patients may often be altered, severely distressed, or critically injured. Such scenarios frequently preclude patients from participating in ocular exams without the use of moderate or general anesthesia [5]. Nonetheless, prompt diagnosis and formal ophthalmology referral remain of critical importance as delayed referral and operative planning has been correlated with poorer visual outcomes [6,7].

Ocular US has proven a useful tool for diagnosing a multitude of traumatic ocular pathologies [8]. The potential of ocular US to diagnose acute traumatic cataracts was first demonstrated in a 2003 case series. A patient with known, penetrating globe trauma and ocular cataract underwent 20 MHz ocular US. This again demonstrated cataract as well as intact posterior capsule [9]. Since this case occurred, the utility of ocular US as it pertains to traumatic cataracts has been verified and expanded. In a prospective comparative study, 43 cases of known traumatic cataract underwent ocular US prior to intraocular lens (IOL) surgery. Ocular US was not only useful in detecting the presence of cataract, but also highly sensitive for detecting posterior lens disruption [10]. Researchers have also set out to establish a reliable, comparable scale between ocular US image characteristics and cataract opacity on the lens opacities classification scale [11].

## Conclusion

It is estimated that over 55 million people suffer ocular injuries each year. Of these injuries, approximately 1.6 million suffer permanent visual impairment secondary to traumatic cataract [12]. Emergency physicians rely on a relatively static set of tools to diagnose a wide range of ocular pathologies with limited precision. Definitive diagnosis is often not reached until specialist consultation. Only in recent years has ocular US become recognized as a useful diagnostic adjunct in the setting of ocular trauma. In one study, emergency physician-performed ocular POCUS sensitivity and specificity for recognition retinal detachment, vitreous hemorrhage, lens dislocation, globe foreign body, and retrobulbar hematoma were comparable to CT and ophthalmologist exam [13]. Of the numerous pathologies recognizable by POCUS, traumatic cataract was recently added to this list [1].

With this case report, we chronicle an instance of acute traumatic cataract diagnosed by emergency physician-performed ocular POCUS. Although further validation is needed, we postulate that when performed and interpreted by capable physicians, ocular POCUS is exceedingly useful for assessing lens integrity in the setting of ocular trauma.
